# Supplementary material for: Chromothripsis during telomere crisis is independent of NHEJ, and consistent with a replicative origin
Source: Genome Res. 2019 May;29(5):737–49. doi: 10.1101/gr.240705.118 (PMC6499312; doi:10.1101/gr.240705.118)
Supplement: Supplemental Material [file supp_gr.240705.118_Supplemental_file_1.zip › contigs/annotated_contigs/DB51/contig.3.DB51_length_665_mean_cov_11.9684210526.docx]

**DB51_length_665_mean_cov_11.9684210526**

```
AGTAAAGATAGTTTTCATATACCATAATTATTGGGCATTTATTATGGATTGGGAAGTTATCCTTGTGGTTAGTATCTTTCATTGTTGTA
 >chr18:69194838-69195086 + E=2e-137
ATACATTTATCATACTTAATGAACCACAATTGAATATCATTATTAACTAACATCCGTACTTGATTCAGGTTTCCTTGGATTTTACCTAA

TGTCCTTTTTCAGTTTCTGTATCCCATACGGTATACCACATCACATTTAGTTGCCGTGTATCTTGACTGT|TCAGTCAAGTGTTACTTG

AAACACTTGAAACAAGTG|TTTTCAAGAAAACACTGGCAAACTGCCATCAGCACATCAAAAAGCTTATCCACCATGATCAAGTAGGCTT
                   >chr18:69197074-69197237 + E=3e-83 p=9e-03
CATGCCCAGAATGCAAGTTTGACTCAACATACACAAACCAATAAATGTGATTCATCATATAAACAGAACTAAAGATAAAAAC|CACACA
                                                                                   >chr6
TGCAG|TGAGAAGTGAAGCCAGGATTCAACCCAGACTGTTTGACTCCATTATGCTATGCTTTATTCCCAGTAAACTGAATTTATTAATA
:79768077-79768306 - E=5e-126
ATAACACTTGGCTACTCAAGAAAATTGGTGTTAGTCATTTTTTGTAAAAGGGAACCAAATATTTATAAAATCAAATATAATATTTTAGG

CCACGTCGTTGATGGTCAGATTAGTAAATACTGCTGACAAGGAGAG
```
